# “Conferring Authorship”: Biobank Stakeholders’ Experiences with Publication Credit in Collaborative Research

**DOI:** 10.1371/journal.pone.0076686

**Published:** 2013-09-30

**Authors:** Flora M. A. Colledge, Bernice S. Elger, David M. Shaw

**Affiliations:** Institute for Biomedical Ethics, University of Basel, Basel, Switzerland; Université de Montréal, Canada

## Abstract

**Background:**

Multi-collaborator research is increasingly becoming the norm in the field of biomedicine. With this trend comes the imperative to award recognition to all those who contribute to a study; however, there is a gap in the current “gold standard” in authorship guidelines with regards to the efforts of those who provide high quality biosamples and data, yet do not play a role in the intellectual development of the final publication.

**Methods and findings:**

We carried out interviews with 36 individuals working in, or with links to, biobanks in Switzerland, in order to understand how they interpret, apply and value authorship criteria in studies involving biosamples. The majority of respondents feel that authorship is an important motivating factor in working and publishing collaboratively. However, our findings suggest that in some cases, authorship guidelines are being ignored in favor of departmental standards which recognize “scientific work” as meriting authorship.

**Conclusions:**

Our results support the current calls in the literature for an alternative method of crediting biomaterial contributions, in order to ensure appropriate authorship inclusion and promote collaborative research involving biobanks.

## Introduction

In recent years, there has been a steady increase in clinical research involving large numbers of collaborators, often spanning multiple departments and research centers, sometimes between several countries. This is partly due to the growing importance of translational research, whole genome studies, and biobanks [1]. It is now possible, and even necessary, for researchers to pool resources from around the globe, either by sharing clinical or genetic data, or by sending physical samples to one another [2]. Consequently, many individuals are involved in some phase of these studies, and their contribution must be acknowledged in the final stage of the research process: publication [3].

Being credited as an author on scientific articles is an essential part of a researcher’s career [4]. In some countries it is also a yardstick by which academic departments are assessed and awarded funding [5]. Coupled with the above-mentioned “team sport” nature of current research, it is not surprising that the increase in multi-collaborator studies has been matched by an increase in authors on published articles [6]. Author lists of several dozen names are now commonplace; some papers have hundreds [7,8]. In light of this, even those accustomed to the norms of scientific research have raised eyebrows about how so many contributors can be said to have had a hand in authoring a single work [9].

The criteria for authorship developed by the International Committee of Medical Journal Editors (ICMJE) have stood for almost thirty years, with periodic revisions, and are adhered to by the majority of biomedical journals (some of which also have their own detailed standards.) [10] They state that authors must make a “substantial contribution” to the conception, analysis or obtainment of the material, the drafting or revision of the manuscript, *and* approval of the final version. The goal is to ensure that any individual listed as an author can defend the work [11]. However, bending and breaking of these rules is widely reported [12,13]; it is frequently taken as given that certain authors on a paper may have made only a few comments, or scanned a draft. Jostling for a place (and particularly a prestigious place) on the author list can lead to bickering and, in some cases, significant career setbacks, especially for those in dependent positions who lack negotiation authority.

Projects involving biosamples from multiple sources add another complication: how to credit people who have provided essential materials, but have not necessarily contributed significantly to the analysis or reporting that followed [14]. In such cases, the research could not have taken place without the contribution of these individuals, who nonetheless do not meet the full ICMJE criteria. To credit such contributors as authors would therefore violate the current guidelines if they are interpreted in the stricter sense. This potentially creates a problem for the development of biobanking, in that those who manage and provide samples might feel they are not receiving sufficient recognition for their work if the authorship criteria are respected [15]. This issue has been neglected in the biobanking literature, and our results reveal some important findings on this topic.

## Methods

### Ethics statement

The study protocol was submitted to the local cantonal research ethics committee (Ethik Kommission Beider Basel) and we received a positive answer after an expedited process (minimal risk study not involving patients). The ethics committee did not require written consent to be obtained for the competent, non-vulnerable individuals who took part in this non-clinical study. Verbal consent was therefore obtained and recorded at the beginning of each interview.

### Study protocol

Semi-structured interviews were carried out with individuals working in, or with close links to, biobanks based in Switzerland. Using purposive sampling, we aimed to identify appropriate individuals through author lists on publications, biobank and academic networks, professional contacts, and a snowball approach. Biobank managers, pathologists, researchers, clinicians, lawyers and ethicists were all identified and approached, first by initial and follow-up email, and in the case of non-response, by telephone. Depending on the convenience of the interviewee, interviews were then arranged either in person or by telephone. The interviewer followed a semi-structured interview guide (see details on development below), and interviewees were informed that they should feel free to introduce issues not addressed by the interviewer. Confidentiality was granted; subsequent transcriptions were fully anonymised in order to prevent identification through names or recognizable situations.

The interview guide was developed in tandem with a literature review on current roadblocks to wide biosample sharing. Issues identified in the literature informed the key question areas, following a brief section to obtain demographic data. Questions regarding authorship were posed in the context of a broader interview guide which addressed other biobanking activities. Questions covered the motivating effect of authorship, interviewees’ perceptions and experiences with current authorship arrangements, and possible problems with the status quo.

Following each interview, verbatim transcriptions were made. Upon completion of the interview process, these were analyzed by four members of the research team, including all authors. Content analysis and coding, following classical qualitative methodology [16], was carried out independently in order to develop themes and sub-themes. These were then compared amongst four team members, including all authors. The authors then agreed upon the themes for this article. The qualitative methodology we employ is based on the model outlined by Mayring in “Qualitative Content Analysis”, employing first inductive development, then deductive development, of themes. Due to the relatively small sample size and nature of our interview guide, we did not develop a model to categorize our findings beyond the general subject groupings we present in the text. Instead, we opted to present broad themes using quotations extensively, in order to give a descriptive overview of our findings, rather than seeking to quantify particular response categories.

## Results

70 stakeholders were approached; 36 of these agreed to be interviewed for our study (17 face-to-face, and 19 by telephone). Amongst those who did not participate, 25 could not be contacted by either phone or email; the remaining nine replied with a refusal, stating either that they did not have time to participate, or that they felt that they could not be helpful in answering our questions, based upon an introductory description of the study. In each instance of non-participation, we sought to identify stakeholder with similar professional qualities and biobank affiliation. Participants include seven biobank managers, three lawyers or ethicists working in the field of biobanking, two administrators, and numerous clinicians from various disciplines. A small number work for private organizations, with the majority employed by an academic institution. Our group contains Swiss, British, Swedish, Italian and German nationals. The majority of interviewees have also worked and/or trained abroad for several years.

In all but two cases, interviews were carried out in English in order to ensure comparability. Exceptions were made for individuals who felt more comfortable expressing themselves in their mother tongue. Consequently, one interview was carried out in German and one in French. Interviews lasted between 30 minutes and one hour.

### Authorship as motivating factor

The great majority of those interviewed agreed that authorship on a publication was indeed a motivating factor in collaborative research and sample sharing, although none stated that it was the chief factor. A number of different reasons as to why authorship credit is such a strong motivator were cited. Prestige in publishing in a well-known journal was often mentioned, with some respondents noting that this was important for institutions as well as individual careers: “…there’s another opportunity to put your laboratory in a bigger paper that will make *Nature* again, then you share the samples.”(I35). Another noted that the influence of funding bodies was a factor: “…it’s very important for you to have, let’s say, a first authorship in a very good journal, because that will help you to get money for research in the next round of grant applications…”

While “publish or perish” is the oft-cited mantra of university departments, our respondents also emphasized that non-academic institutions have an interest in publication credit: “…for example, we are not a university institute, but it’s nice for us to be part of a publication which is visible…” (I22); “…for some, let’s, say gastroenterologists in private practice, that’s quite an achievement, they see publication with their name on it even indirectly, and they can, show it in their private practice…” (I15).

Two respondents also stated that authorship acts as an alternative motivator to financial compensation for sample use: as one put it, “Authorship is a kind of payment.” (I6)

However, a sizeable group stated that authorship was not a motivating factor, or at least not the most important one, in encouraging sample exchange. The small number who suggested that authorship did not motivate collaboration at all stressed that this would be an inappropriate focus for biobank stakeholders and researchers. This sentiment was echoed among the slightly larger group who felt authorship was simply not the main motivation: “I think it’s not always possible, it’s not always applicable, but I think the motivation should … it’s like with money. And I think one should not work for the payment, just having that as prior aim, but this is something you need to go further.” (I6) Respondents felt that answering research questions was the key motivating factor: “It’s not that we just do it for publications, I mean we do it because we want to answer the scientific questions.” (I7)

Although respondents did not discuss it in terms of a motivational aspect, the visibility which authorship brings to the researcher’s biobank was noted several times. When asked about how they identified potential collaborators, respondents pointed towards publications: “…it wasn’t looking for biobanks, and then, but it was more that they publish in an area so you got aware of them.” (I14)

### Criteria for authorship

When questioned about the criteria which must be met to become an author on a paper, our interviewees identified a variety of considerations. A large number felt that authorship was a form of recognizing contribution: “…people … wanted to be recognized for the work they are doing ... And recognition in university, is authorship.”(I18)

Interviewees who held this view of authorship generally stated that involvement in the research process was the necessary element: “If they do some scientific [work] then it’s no question to be a co-author.”(I11)

Several participants described criteria for authorship using numbers and percentages of samples (or patients) contributed relative to the study size: “…if you share ten samples in a biobank that has 500, then they do some research, and they publish something, is it fair that you ask coauthorship, or then you … the only thing was you took ten samples?” (I33) In most cases, they stated that while this method was often employed in some way, it was not clearly established in all cases prior to collaboration, and sometimes led to confusion : “Now this is not always easy, because some senders they give me fifteen patients, others they give me one patient, and then each one of them wants to be recognized, because, someone gave me one patient, four clinicians were involved in that or whatever, and the sender that gave me fifteen patients gives me only two clinicians, so how do I keep … a sense of justice?” (I32) A small number do, however, have a standard policy based on numbers contributed; for example: “And the few, you know, there’s a limited amount of authors that can be listed, so we will also pick those who have the highest amount of samples contributing.”(I28)

While discussion of the criteria for authorship was somewhat vague, some interviewees had strong sentiments about what were not sufficient criteria. In direct contrast to the comments above, several stated that guidelines on authorship do not recognize mere sample contribution as grounds for inclusion: “The criteria to be on a paper, are pretty clearly defined, I think you have to, just to send samples or to be, or recruit patients, that’s not enough.” (I24) There was a marked distinction between respondents who agreed with this standard: “…what is the scientific work to go to the cellar and open a freezer and take out some samples?” (I11); “…publication for me means also exchange of effort between the researcher.” (I6) and those who appeared to recognize the standard, but knowingly ignore it: ”…but that’s just the way it works, the way it works is that if you want their samples you have ... or their patients, you have to. do yourself the work, and be accommodating. You can, maybe you can call this very unfair, but that’s the way the world is.”(I27); “…I cannot contribute, because I need the data to … say something. And then I said it’s nice … if you want to keep me as a co-author it’s ok, but. and then we come back to the criteria for the co-author, you should be involved and you should know what they [other authors] say [in the paper].”(I33)

### Difficulties

Biobank stakeholders we spoke to reported experiencing certain problems directly related to authorship arrangements. In view of the comments above, it is interesting to note that a few interviewees disagreed strongly with the suggestion that providing samples should not lead to authorship. Broadly, the notion that contributing samples is “mere” administrative work was contradicted: “Absolutely no. Absolutely no. This is … so … underestimated, our effort. So you need a lot, a lot of time, manpower also.” (I29); “…and we always fight to being credited when biological samples are used, so that … some of the researchers who join later, the project, who are just not aware what it had cost to, to get all these samples organized, they think it’s just available to be used…”(I14)

Beyond the issue of whether or not collaborators deserve authorship for a given contribution, several respondents stated that arguments over inclusion and position do occur, sometimes with serious consequences: “I’ve seen so many friendships destroyed because of authorships, and I don’t want to be part of that.” (I32)

The most frequently mentioned grievance in such disagreements was the use of rank, or hierarchy, to determine authorship, rather than man hours: “…big boss in one lab that I know well signed the paper, the guy who had the initiative and the idea, and the postdoc who did the work was in the acknowledgments. Big boss wants his name on it … if he’s a schmuck he’s a schmuck.” (I32)

Even amongst respondents who had not experienced any difficulties, personal relationships, in particular the influence of the lead investigator, were noted as being instrumental in establishing authorship: “I think it’s fair in this department, I think it’s a culture that the boss creates, whether it is fair or not.” (I9)

Interestingly, one respondent brought up the possibility of refusing an offer of authorship due to disagreement with the use of their samples: “…even if this person would pay me a lot, I, I would not agree. If this center would say, “You are going to be on the publication” and I cannot trust what they’re doing … it did even happen during the years that I refused to have my name.” (I6)

### Numerous authors on science papers

Finally, several of our interviews brought up the topic of the ever-increasing number of authors on scientific papers. Those who had been working in the field for a long time noted that this phenomenon is relatively new: “I was struck by the fact that [a few decades ago] maybe on 5% of scientific papers has co-authors…” (I34). Another described the discussions about assigning credit in such cases as “…like a souk … I’m not going to fight with 179 other co-authors…” (I32) Despite the potential for conflict in such a situation: “…you put together your samples, and you do a genetic study, then you identify something, a publication comes out, and it is clear that not everybody can be a first author or last author.”(I30) All those who discussed the issue were ambivalent about the development: “…if you have a publication, and, and you’re the author number thirty-two, of fifty-five, what is the value of that? You can publish in *Nature*, it’s still nothing.” (I11) It was also suggested that this ambivalence might be a reflection of individual career status, rather than the lack of value in being one amongst many authors: “…these researchers, they are very advanced … they don’t care that much if they are 1 in 300 authors because they have the name already.” (I35)

## Discussion

To our knowledge, this is the first qualitative study which has sought to describe and analyze the experiences and perceptions of biobank stakeholders regarding the attribution of authorship in research using human tissue and/or data. As tools for biomedical research, biobanks are also by default tools for publishing findings, and authorship is a crucial aspect of professional life for the majority of individuals working in connection with biobanks. In multi-center, population-wide or transnational studies, the number of individuals with a stake in having their name on a paper may be very high. In view of the difficulties associated with publication credit, our results provide valuable insight into how affected individuals perceive, and deal with, current practices.

Our interviewees were nearly unanimous in agreeing with the proposition that authorship is a motivation to make samples available to other researchers, or collaborate with individuals external to their own department in some way. However, a significant number stated that it was not their main motivation, and several respondents made comments to the effect that chasing authorship could in some cases be a distraction from research itself. Given that this latter attitude may be a more socially desirable one, it is interesting to note how many respondents were ready to admit that authorship was in fact a strong motivator.

Several interviewees also expressed ambivalence about being included on a long list of authors, and stated that this provided few of the above-mentioned career benefits. Indeed, senior authorship seemed to be the most important motivating factor for researchers working at universities, although others found authorship credit as such motivating. Our interviewees also addressed other issues which are prevalent in the literature, such as disputes regarding position and the influence of hierarchy, which are generally accepted as unfortunate but predictable in the course of academic research publication[17,18].

Our most interesting finding is the respondents’ views regarding criteria which do, or do not, qualify an individual to appear on the list of authors. As noted above, the ICMJE has three conditions which must be fulfilled by all authors. It is therefore striking that not only were these conditions, and the document itself, never explicitly referred to; in a number of cases, interviewees adhere to systems which differ significantly. In particular, several interviewees stated that contributing samples (usually above a certain number), or providing some kind of “scientific” input, would be grounds for inclusion as an author (indeed, this is a requirement in some material transfer agreements, such as that of the Chernobyl biobank[19]). In some instances, the policy of basing authorship on sample contribution was described, and then questioned or objected to, by the same individual. This indicates a certain acceptance of such conditions as being just “the way it works”, an attitude which supports the literature suggesting that disregard for, and unawareness of, the ICMJE criteria is widespread[3,5,20,21].

Our findings indicate that there seems to be a more or less variable culture of attributing authorship that goes beyond the present ICMJE criteria. It is important to stress that what respondents in our study describe is not classical “guest” or “gift” authorship, as the biosample contributions were time-consuming and included significant scientific and organizational work. Clarification of rules and transparency of the types of contributions is of utmost importance, as significant diversion from the guidelines has potentially serious consequences[22]; furthermore, systematic disregard for guidelines will devalue them[23]. Several interviewees in our study described policies which seem to contravene a strict interpretation of established guidelines in the sense of both over-inclusion and under-inclusion of authors. Misattribution of authorship is at best “research misbehavior”,[24] at worst research misconduct, and can lead to negative consequences for those involved[25]. Not being credited as an author may hinder an individual’s chances of promotion or obtaining future research grants (this may also be the case if a researcher’s named is pushed further down the list solely due to lack of seniority)[26]. Furthermore, inclusion as an author on a paper which is later revealed to have flawed or questionable findings may be just as detrimental as being omitted from the list of an important publication[27]. Our interviewees indicate that both excessive and insufficient crediting currently occurs, in accordance with the findings of Glänzel and Schubert, who note that certain studies show a tendency to under-acknowledge in-house collaborators, while collaborations with other departments are more thoroughly credited[28,29]. Nevertheless, the tacit acceptance of disregard for these criteria remains a troubling aspect of work with human biological samples.

### Implications

Although not all participants felt that material contribution merits authorship, and some directly contradicted this notion, the decisive element in the disagreement appears to be the amount of effort required to make samples available, rather than correspondence with the ICMJE’s three criteria. This suggests that authorship is currently viewed as the only valuable method of rewarding and recognizing significant professional effort, despite the fact that not all collaborators may be involved in “authoring” the final work. Respondents also emphasized that publication credit is not only important for its traditional influence on individual careers, but is also a form of promotion for the biobank itself. In the absence of standardized methods of biobank accreditation and indexing, the presence of a member of staff on the author list increases the chances of the biobank being contacted by external parties.

There is no provision in the ICJME guidelines which reflects the reality of the evolution of research that necessarily implicates multiple centers and collaborators. Some universities, journals and organizations provide their own guidelines which are more adapted to crediting material contributions: the Swiss Academy of Medical Science, whose directives apply to all our respondents currently, directs authors to their various university guidelines, and provides a list of authorship criteria similar to that of the ICMJE, but requiring that only one condition, rather than all, be satisfied[30]. The University of Basel states that authors should “have made a substantial personal contribution to the planning, execution, evaluation, or supervision of a given research publication […]”, a standard which encompasses the work involved in providing biosamples, with or without accompanying data[31]. However, the very fact that institutional guidelines differ from the international standard is troubling, as it is likely to confuse researchers, particularly those involved in inter-departmental research. This issue is not confined to Switzerland; the multiplicity of subtly different guidelines from organizations around the globe does little to improve an already complicated matter.

Although some biobanks have a policy of only requiring a mention in the acknowledgment section[32], it is possible that researchers involved in work with tissue samples feel obliged to designate important collaborators as authors in the absence of a universally recognized system for rewarding scientific and material contributions. To achieve this, they may take a broad view of the ICJME guidelines, accepting that small changes to the draft, and a final reading of the article, satisfy a relaxed interpretation of the requirements. It seems that an addendum to the guidelines which takes into account the possibility of “scientific work” as fulfilling the criteria of study design and creative contribution is now imperative. While some flexibility in guidelines to allow for individual discretion is advisable, a clearer definition of what constitutes “scientific work” is required to avoid continued misappropriation of author credit, and to enable biobank stakeholders and researchers be acknowledged in a meaningful way.

Several alternatives currently exist, or have been proposed, which recognize contributions outside of the traditional authorship framework : these include acknowledgment sections, contributorship statements such as those pioneered by the British Medical Journal[33], the Biological Resource Impact Factor[15] and the ORCID (**http://www.orcid.org/**) initiative to permit recognition of both bioresources and stakeholders. The latter two are particularly promising prospects for biobank stakeholders, as they are specifically tailored to the challenge of acknowledging sample contribution (important for individuals), yet also to providing visibility and endorsement for sample collections (important for the biobank as a research entity). Suggested methods include assigning biobank unique identifying numbers which can then be used to credit banks that provide resources which lead to publication, and standardized, universally employed recognition of biobank employees in the methods section of articles. Universal adoption of such a system will be a key step in both resolving authorship issues and promoting biobanks as indispensable research resources. It may also be illuminating to look to other disciplines for inspiration. Dozens of particle physics papers, for example have over three thousand authors[34]. One collaborative team, in 1998, pioneered a novel set of standards for authorship on all publications issuing from the group, requiring all authors to have worked at the lab for a year, although independent of the direct input of that individual on any particular project. The existence of a"highly bureaucratic internal structure, small size, people doing tasks and thinking together in the same site"[35] means that such authorship criteria, while not traditional, are an adaptive means of coping with evolving research norms.

### Limitations

Our study has some limitations. First, authorship is a very sensitive issue and in spite of anonymity interviewees might not have felt secure enough to speak openly. In addition we expect a bias towards social desirability. It is therefore a strong finding that some interviewees admitted openly to be motivated very much by publications. We believe that it is not a significant limitation that interviewees were recruited in Switzerland because there is no reason that their opinions would vary significantly from researchers of other Western countries. Most of the interviewees have international experience and work in international collaborations. None of them reported a different “Swiss way”, but referred to an international authorship culture they encountered as part of their multiple collaborations.

## Conclusion

Authorship continues to be a benchmark by which researcher’s careers are measured, yet the guidelines for its attribution are frequently disregarded, in some cases due to ignorance of their very existence. As multiple-author papers proliferate, so too do the problems associated with them. Biobanks are a chief source of the collaborations which produce such papers. Our interviews with biobank stakeholders in Switzerland reveal that authorship is considered a motivating factor for collaborative research, but that there are numerous instances of inappropriate credit and dispute. A main factor in this may be the lack of a suitable alternative method of recognizing the essential contributions of those who provide well-annotated, high-quality bio-specimens, but may not contribute to the intellectual development of resulting articles. In order to maintain the integrity of the authorship system, and encourage the evolution of biobanking, a suitable system of crediting authors must be agreed upon by researchers and journals.
